# SUBJECTIVE AGE MODERATES THE RELATIONSHIP BETWEEN GLOBAL COGNITION AND SUSCEPTIBILITY TO SCAMS

**DOI:** 10.1093/geroni/igad104.1625

**Published:** 2023-12-21

**Authors:** Gali Weissberger, Aaron Lim, Laura Mosqueda, Annie Nguyen, Laura Fenton, Duke Han

**Affiliations:** Bar Ilan University, Ramat Gan, HaMerkaz, Israel; University of Southern California, Los Angeles, California, United States; Keck School of Medicine of the University of Southern California, Los Angeles, California, United States; University of Southern California, Los Angeles, California, United States; University of Southern California, Los Angeles, California, United States; University of Southern California, Los Angeles, California, United States

## Abstract

Understanding factors that increase susceptibility to scams in old age is of critical importance. Cognitive functioning has been demonstrated to be an important factor in predicting financial exploitation and scam risk. There is also evidence that subjective age, or one’s perceived age relative to chronological age, may be associated with financial exploitation risk. This study examined the interactive effect of subjective age on the relationship between global cognition and susceptibility to scams. Sixty-five participants underwent an assessment of global cognition (Mini-Mental State Examination; MMSE), reported their subjective age, and responded to a self-report questionnaire on scam susceptibility. A main effect of global cognition on scam susceptibility was found (b = -0.029, ß = -0.280, p = 0.028); there was no main effect of subjective age on scam susceptibility (p = 0.819). An interaction between cognition and subjective age was found, such that the negative association between cognition and scam susceptibility increased as subjective age increased (b = -0.185, ß = -0.311, p = 0.016). Examination of conditional effects demonstrated that the relationship between cognition and scam susceptibility was not significant amongst those with subjective ages below one standard deviation (SD) of the mean, but was significant for those whose subjective ages fell around or above the mean (within 1SD, b = -0.03, SE = 0.01, p = 0.03; +1SD, b = -0.06, SE = 0.02, p = 0.001). Findings suggest that individuals with older subjective ages may be particularly vulnerable to the negative effects of lower cognition on scam susceptibility.

